# On Modern Progress in Ophthalmic Medicine and Surgery

**Published:** 1894-06-02

**Authors:** Robert Brudenell Carter

**Affiliations:** Consulting Ophthalmic Surgeon to St. George's Hospital


### On Modern Progress in Ophthalmic Medicine and Surgery.

By Robert Brudenell Carter, F.R.C.S., Consulting Ophthalmic Surgeon to St. George's Hospital.

A patient about to undergo a cataract operation should be placed flat on his back, upon a couch of convenient height, sufficiently narrow to permit of easy access to the face from either side. The foot of the couch should be close to a window, and its length at right angles to this window, so that light may fall upon the face; but the time of the day, in relation to the position of the window, should be such as to avoid direct sunlight. The head should be supported by a small and rather firm pillow, so as to render the plane of the face nearly, but not quite horizontal, the chin being a little inclined towards the chest. The conjunctival secretions, and any residues of the cocaine wafers, having been washed away in the manner already described, the upper lid should be gently engaged in the loop of a Noyes speculum, and raised by an assistant, who, for an operation on the right eye, should stand on the right of the surgeon, and for an operation on the left, upon his left; the hand holding the handle of the speculum passing between the two hands of the surgeon, and being carefully kept out of his way. The assistant must watch the position of the speculum, so as to see that it raises the lid sufficiently, and that it does not touch the eyeball. When this is arranged, the surgeon, standing at the end of the couch behind the patient, should take the knife in his right hand for the right eye and in his left hand for the left. Perfect ambidexterity, as far as the use of the knife is concerned, is essential to good operating; and any surgeon who cannot attain this ambidexterity will do well to direct his attention to some other department of the healing art. The lower lid should be gently drawn down by a finger of the hand which holds the knife, and a vertical fold of conjunctiva and sub-conjunctival tissue, just below the lower margin of the cornea, should be pinched up with fixation forceps. These forceps should be rather broad, with at least four teeth in one blade and three in the other, and they should present to the surface of the eye a flat circular extremity at least two millimetres in diameter, and preferably somewhat more. Forceps smaller than this are apt to tear the conjunctiva, and thus to lose the security of their hold at a critical period of the operation. Young operators often experience difficulty in attending equally to the course of the knife and to the position of the forceps; and are apt, while watching the former, unconsciously to exert pressure or traction with the latter, a course which may easily be fraught with injurious consequences, and which no pains should be spared to prevent.

The knife used by many operators, and called Von Graefe's, has a blade three centimetres in length and two millimetres or a little more in width; but, having found this pattern more convenient when reduced by grinding, I now have the blade made only one millimetre in width, or as narrow as possible. Secure fixation being attained, the point of the knife is entered on the temporal side of the anterior chamber, behind the true cornea and in front of the iris, usually at a spot about one millimetre below a horizontal line tangential to the upper margin of the cornea. The point receives at first a slight inclination downwards, but is then made to traverse the anterior chamber horizontally, and to emerge by counter-puncture at a point opposite to and corresponding with that of entrance. Until counter-puncture is completed, it is necessary to press the back of the blade against the corneal tissue, in order to retain the aqueous humour by avoiding any enlargement of the wound of entrance. As soon as the counter-puncture is completed, the edge should press against the corneal tissue as the blade is pushed on, so as to divide a portion of the bridge between the punctures; and this division should be completed by a to-and-fro movement of the blade, which should cut only in advancing, and be loosened in the incision as it is drawn back. At the same time the edge should be directed forwards, and the incision as a whole should curve gently, in such a degree that its centre just coincides with the margin of the true cornea. Some surgeons prefer to keep it farther back, so as to form a flap of conjunctiva, but this course appears to me objectionable rather than otherwise. The edge of such a flap seldom falls into perfect contact with the portion from which it has been severed, and is apt to swell and to become a source of irritation during the progress of healing. The narrowness of my own pattern of knife much facilitates the forward direction of the edge, because a broad blade, when turned in this way, is apt to press upon the iris, and through the iris upon the lens, in a manner which may even lead to rupture of the hyaloid membrane.

As soon as the incision is completed the speculum may be removed and the upper lid suffered to fall. After a brief interval it is gently raised by the operator, and any drop of blood which may be present is removed by a touch with a morsel of aseptic sponge, squeezed out of the boroglyceride solution. The next step is the performance of iridectomy.

For this part of the operation no speculum is necessary. The surgeon can raise the upper lid sufficiently with the third or little finger of his left hand, while he holds the iris forceps between the thumb and index-finger, the patient looking somewhat downwards. The closed forceps are carried into the anterior chamber until their points arrive nearly at the margin of the pupil. They should not dip after the iris, but should be suffered slightly to expand, and the fold which rises between them is seized as they are closed. The patient will sometimes feel this step of the operation, and, in order to diminish sensation, it is desirable not to exercise traction as soon as the iris is seized, but to wait a moment before doing so. The fold of iris should then be slowly drawn out of the anterior chamber, and divided with scissors on the right of the forceps, that is, at the temporal end of the incision for the right eye, and at the nasal end for the left. The piece held by the forceps, and severed from the main circle on one side, must be torn from its peripheral attachment up to the other extremity of the wound, and there cut off by the scissors in such a way that the remainder will retract into the anterior chamber. It is a matter of supreme importance that no portion of iris should be left in such a position as to become engaged in an angle of the wound.

